# The health of the residents of Ireland: Population norms for Ireland based on the EQ-5D-5L descriptive system – a cross sectional study

**DOI:** 10.12688/hrbopenres.12848.1

**Published:** 2018-09-04

**Authors:** Anna Hobbins, Luke Barry, Dan Kelleher, Ciaran O'Neill

**Affiliations:** 1 Centre for Public Health, Queens University Belfast, Belfast, UK; 2 J.E. Cairnes School of Business and Economics, National University of Ireland Galway, Galway, Ireland

**Keywords:** EQ5D, Ireland, Population norms, disparities, Socio-economic status

## Abstract

**Background:** The EQ-5D descriptive system has become a widely used generic instrument to measure population health. In this study we use the EQ-5D-5L system to describe the health of residents in Ireland in 2015/16 and examine relationships between health and a range of socio-demographic characteristics.

**Methods:** A representative sample of residents in Ireland was established in a two-stage random sampling exercise in 2015/16. Self-reported health, together with a range of socio-demographic characteristics, were collected using a computer-assisted-personal-interview survey. Self-reported health was captured using the EQ-5D-5L descriptive system including a visual analogue scale. Data were presented as descriptive statistics and analysed using a general linear regression model and ordered logistic regression models in the case of specific health domains. Socio-economic gradients in health were also examined using concentration curves and indices.

**Results**: A usable sample of 1,131 individuals provided responses to all questions in the survey. The population in general reported good health across the five domains with roughly 78%, 94%, 81%, 60% and 78% reporting no problems with mobility, self-care, usual activities, pain/discomfort and anxiety/depression respectively. Differences in health with respect to age, and socio-economic status were evident; those who were older, less well-educated of lower income and without private health insurance reported poorer health. Differences in health between groups differentiated by socio-economic status varied across domains of health, and were dependent on the measure of socio-economic status used.

**Conclusion: **Residents of Ireland appear to rate their health as relatively good across the various domains captured by the EQ-5D-5L system. A pro-affluent gradient in self-reported health is evident though the sharpness of that gradient varies between domains of health and the measures of socio-economic status used. The study provides baseline data against which the health of the population can be measured in the future as demography and economic conditions change.

## Introduction

Central to the development of effective health policy is an ability to measure health and any changes in it that attend novel healthcare or policy interventions. While a variety of instruments exist to measure disease specific aspects of health ^1^, generic measures can be useful when a broader perspective is required, allowing comparison across many diseases, for example ^2^, or when attempting to inform the allocation of healthcare resources ^3, 4^. The EQ-5D descriptive system ^5^ has become a widely used generic measure of health, with a large number of studies using this to describe the health and changes in health overtime of different groups ^6– 11^. The EQ-5D instrument describes health across five domains: mobility, self-care, usual activities, pain/discomfort and anxiety/depression. While originally measured at three levels with respect to each domain – the 3L version of the instrument ^5 ^- more recently a 5L version of the instrument has been developed ^12^ where health is measured at five levels. The 5L version has been shown to have fewer ceiling effects and to have greater discriminatory power ^13– 17^ than the 3L version.

Using the EQ-5D-5L descriptive system it is possible to describe 3125 (5 ^5^) unique health states based on combinations of levels in the different domains. Health states can be described as a crude sum score in which the scores of the five domains (values 1–5) can be added (sums between 5 and 25), from which 5 is deducted (range 0–20), and the result multiplied by 5 to give a range between 0 and 100; this value is then deducted from 100 so that higher values represent better health on a scale from 0 to 100. The descriptive system can also be combined with preference weights for the health states to produce a health utility index reflecting relative preference for health states. Preference weights are generated from valuation exercises of representative samples of the country concerned, 5L preference sets existing for 11 countries at the time of writing ^18– 28^. 

Using the 5L system, studies have produced descriptions of population health for representative samples of the population in Germany ^29^, England ^17^ the USA ^30^, Poland ^31^, South Korea ^32 ^ and Vietnam ^33^. These studies have also examined differences between sub-groups of the population related to age and gender showing broadly consistent patterns in which older people and females exhibit poorer self-reported health relative to younger persons and males.

The development of a 5L value set for Ireland in 2015/16 afforded the opportunity to use the 5L descriptive system to measure the health of a representative sample of residents and examine differences in health between distinct sub-groups. In this paper we present the methods and results of this study. As the Irish healthcare system is a mixed public/private system with respect to both finance and provision, socio-economic status can be an important determinant of access to services. In the paper we explore differences in health, related to socio-economic status including possession of private health insurance (PHI). This is to the best of our knowledge the first study of its type in Ireland. To encourage further work in the area the data upon which the study is based have been deposited with and are freely available from the Irish Social Science Data Archive.

## Methods

### Ethical considerations

Ethical approval for the study was granted by NUI Galway Ethics Committee (application number 15/JAN/04). Written informed consent was gained from all participants.

### Sample

As noted, the study was conducted alongside the generation of a value set for Ireland and study design was therefore informed by the protocol used for the construction of the value set. Following Oppe and van Hout (2017) ^34^ a representative sample of at least 1000 respondent preferences was required for the value set study. The sample was generated using a two-stage stratified clustering process as detailed by Haase and Pratschke, (2012) ^35^. In the first stage, a sample of 54 small areas stratified by deprivation and urban/rural classifications were drawn at random from across the country. *Small areas* are geographical constructs developed by the Central Statistics Office that comprise a minimum of 50 households with a mean of just under 100 and for which population statistics are published. In the second stage, within each small area, a sample of approximately 20 houses were selected at random. Random selection was achieved by using a random starting point and inviting a resident in every third house to participate in the survey. To achieve this, each house was visited up to three times throughout the day and evening in an effort to make contact with the householder. Where it was not possible to elicit a response from the selected house, it was replaced from among those in the immediate vicinity. Within each house any adult (>17 years) capable of giving informed consent could volunteer to complete the survey, one volunteer per household being chosen at random.

Interviews were conducted by one of a seven-person team of trained surveyors between March 2015 and September 2016. The interviewer team comprised three males and four females. Each surveyor was trained prior to deployment; training included the conduct of test interviews to ensure the surveyor was competent in use of the survey instrument. On completion of the random sampling exercise, over-representation of older people and females was found in the sample. The sample was therefore augmented with 102 additional respondents selected purposively based on age and gender characteristics. A quality control process developed by the EuroQol Group in which survey work was reviewed on a weekly basis provided independent oversight of the study.

### Survey

Respondents were presented with the EQ-5D-5L survey, which was completed on the interviewer’s laptop using a computer assisted personal interview (CAPI) survey approach. In addition to providing information using the 5L descriptive system, respondents recorded their current health using a visual analogue scale in which health ranged from 0 to 100, with 0 being the worst possible and 100 the best possible imaginable health. Respondents also furnished information on their age, gender, marital status, how many dependent children they had and whether they had a long standing illness. Respondents also furnished details of their income and possession of private medical insurance and medical card status. Medical card status in Ireland refers to whether or not an individual has access to publicly funded GP services free at the point of use as well as prescription medicines. With the exception of certain groups – e.g. children with cancer - access is means tested though different income thresholds apply to those aged 70 and over compared to others. A total of 1,131 EQ-5D-5L surveys with complete socio-demographic data were collected during the survey – Supplementary File 1. (An additional 29 participants provided preference data but not complete socio-economic data; these 29 do not feature in the analyses presented here.)

### Analyses

We calculated the following descriptive statistics for the sample: the proportion of the sample across various socio-demographic characteristics and how this related in each case with respect to the population at large as based on estimates produced by the Central Statistics Office ( Table 1); the percentage of the sample at each level of each domain as well as by age group (18–24, 25–34, 35–44, 45–54, 55–65, 64–75, and 75+ years) ( Table 2) and by gender ( Table 3) to facilitate comparative analysis.

**Table 1.** Socio-demographic characteristics.

Socio demographic	Sample N= 1,131		General Population of Ireland 2011
		%	%
Marital status			i
Married/living as married	680	60	50
Never Married	267	24	39
Divorced/Separated	93	8	6
Widowed	91	8	5
Gender			
Male	426	38	49
Female	705	62	51
Location			
Urban	656	58	62
Rural	475	42	38
Dependents U18 years			
Living with Dependents under 18 years	444	39	42
not living with Dependents under 18 years	687	61	58
Age			
18–25	88	8	12
25–34	165	15	22
35–44	221	19	20
45–54	229	20	17
55–64	182	16	13
65–74	156	14	9
75+	90	8	7
Ethnicity			ii
Irish	1005	89	83
European (non-Irish)	86	8	12
Other	40	3	5
Economic activity			iii
Employed part-time and full-time	552	49	50
Unemployed	71	6	12
Student	70	6	11
Long-term sickness or disability	42	4	4
Home duties/looking after home or family	124	11	10
Retired	255	22	13
Other (specify)	17	2	0
Education			iv
Primary	86	7	14
Second Level or less	414	37	37
Third level	631	56	43
Not stated/no formal education	0	0	6
Household income €	1131 v		
€0 – €10,000	48	4	
€10,000 – €20,000	157	14	
€20,001 – €30,000	175	15	
€30,001 – €40,000	139	12	
€40,001 – €50,000	119	11	
€50,001 – €60,000	111	10	
€60,001 – €75,000	123	11	
€75,001 – €100,000	127	11	
€100,001 – €200,000	111	10	
€200,000 >	21	2	
Self-rated health using EQ-5D-5L			
11111	516	46	
Any other health state	615	54	
Self-rated health using EQ-VAS			
<80%	386	34	
80–89%	302	27	
90–99%	370	33	
100%	73	6	

i= The full population of Ireland (4,588,252) married not including living as married in CSO figures (adult population, 3,439,565)

ii= Usually resident population by place of birth and nationality = 4,525,281)

iii= The labour force = total population over the age of 15 years is 3,608,662. Total Employed over 15 years (Full-time + Part-time) = 1,807,360. The sample employed also includes (Full-time + Part-time) employed and employed and self-employed.

iv= Total population = 3,003,490 (Population aged 15 years and highest level of education completed)

v= The number of individuals who reported their household income.

**Table 2.** Self-reported health by age.

	Self-reported EQ-5D-5L raw numbers, percentages by age group: Total																
Parameter		Age														Total	
		18–24		25–34		35–44		45–54		55–64		65–74		75+			
		n	%	n	%	n	%	n	%	n	%	n	%	n	%	n	%
Total	N	88		165		221		229		182		156		90		1131	
Mobility	No Problems	79	89.8	158	95.8	205	92.8	183	79.9	130	71.4	93	59.6	38	42.2	886	78.3
	Slight problems	8	9.1	5	3.0	8	3.6	30	13.1	31	17.0	32	20.5	29	32.2	143	12.6
	Moderate problems	1	1.1	0	0.0	7	3.2	14	6.1	15	8.2	22	14.1	18	20.0	77	6.8
	Severe problems	0	0.0	1	0.6	1	0.5	1	0.4	4	2.2	8	5.1	5	5.6	20	1.8
	Unable	0	0.0	1	0.6	0	0.0	1	0.4	2	1.1	1	0.6	0	0.0	5	0.4
Self-care	No Problems	87	98.9	163	98.8	216	97.7	221	96.5	165	90.7	142	91.0	66	73.3	1060	93.7
	Slight problems	1	1.1	1	0.6	5	2.3	4	1.7	13	7.1	8	5.1	22	24.4	54	4.8
	Moderate problems	0	0.0	1	0.6	0	0.0	3	1.3	3	1.6	4	2.6	1	1.1	12	1.1
	Severe problems	0	0.0	0	0.0	0	0.0	1	0.4	1	0.5	1	0.6	1	1.1	4	0.4
	Unable	0	0.0	0	0.0	0	0.0	0	0.0	0	0.0	1	0.6	0	0.0	1	0.1
Usual activities	No Problems	78	88.6	153	92.7	199	90.0	181	79.0	135	74.2	118	75.6	50	55.6	914	80.8
	Slight problems	7	8.0	9	5.5	15	6.8	33	14.4	31	17.0	15	9.6	17	18.9	127	11.2
	Moderate problems	3	3.4	2	1.2	6	2.7	12	5.2	11	6.0	15	9.6	13	14.4	62	5.5
	Severe problems	0	0.0	1	0.6	0	0.0	1	0.4	4	2.2	6	3.8	9	10.0	21	1.9
	Unable	0	0.0	0	0.0	1	0.5	2	0.9	1	0.5	2	1.3	1	1.1	7	0.6
Pain/Discomfort	No	67	76.1	123	74.5	152	68.8	130	56.8	90	49.5	68	43.6	43	47.8	673	59.5
	Slight	17	19.3	36	21.8	47	21.3	51	22.3	52	28.6	45	28.8	22	24.4	270	23.9
	Moderate	4	4.5	6	3.6	21	9.5	38	16.6	30	16.5	34	21.8	19	21.1	152	13.4
	Severe	0	0.0	0	0.0	1	0.5	8	3.5	10	5.5	8	5.1	4	4.4	31	2.7
	Extreme	0	0.0	0	0.0	0	0.0	2	0.9	0	0.0	1	0.6	2	2.2	5	0.4
Anxiety/depression	No	70	79.5	128	77.6	171	77.4	170	74.2	141	77.5	130	83.3	72	80.0	882	78.0
	Slight	9	10.2	23	13.9	32	14.5	35	15.3	30	16.5	16	10.3	11	12.2	156	13.8
	Moderate	8	9.1	10	6.1	16	7.2	20	8.7	9	4.9	9	5.8	7	7.8	79	7.0
	Severe	1	1.1	3	1.8	2	0.9	4	1.7	1	0.5	1	0.6	0	0.0	12	1.1
	Extreme	0	0.0	1	0.6	0	0.0	0	0.0	1	0.5	0	0.0	0	0.0	2	0.2

**Table 3.** Self-reported by age and gender.

	Self-reported EQ-5D-5L raw numbers, percentages by age group: Males																
Parameter		Age														Total	
		18–24		25–34		35–44		45–54		55–64		65–74		75+			
		n	%	n	%	n	%	n	%	n	%	n	%	n	%	n	%
Total	N	52		67		76		76		67		55		33		426	
Mobility	No Problems	48	92.3	64	95.5	70	92.1	64	84.2	45	67.2	31	56.4	13	39.4	335	78.6
	Slight problems	3	5.8	2	3.0	2	2.6	9	11.8	13	19.4	14	25.5	13	39.4	56	13.1
	Moderate problems	1	1.9	0	0.0	4	5.3	2	2.6	7	10.4	5	9.1	6	18.2	25	5.9
	Severe problems	0	0.0	0	0.0	0	0.0	0	0.0	1	1.5	4	7.3	1	3.0	6	1.4
	Unable	0	0.0	1	1.5	0	0.0	1	1.3	1	1.5	1	1.8	0	0.0	4	0.9
Self-care	No Problems	51	98.1	67	100.0	75	98.7	72	94.7	63	94.0	46	83.6	27	81.8	401	94.1
	Slight problems	1	1.9	0	0.0	1	1.3	2	2.6	3	4.5	5	9.1	6	18.2	18	4.2
	Moderate problems	0	0.0	0	0.0	0	0.0	1	1.3	1	1.5	3	5.5	0	0.0	5	1.2
	Severe problems	0	0.0	0	0.0	0	0.0	1	1.3	0	0.0	0	0.0	0	0.0	1	0.2
	Unable	0	0.0	0	0.0	0	0.0	0	0.0	0	0.0	1	1.8	0	0.0	1	0.2
Usual activities	No Problems	47	90.4	61	91.0	68	89.5	61	80.3	47	70.1	41	74.5	21	63.6	346	81.2
	Slight problems	3	5.8	6	9.0	5	6.6	10	13.2	14	20.9	7	12.7	7	21.2	52	12.2
	Moderate problems	2	3.8	0	0.0	2	2.6	4	5.3	5	7.5	2	3.6	2	6.1	17	4.0
	Severe problems	0	0.0	0	0.0	0	0.0	0	0.0	0	0.0	4	7.3	3	9.1	7	1.6
	Unable	0	0.0	0	0.0	1	1.3	1	1.3	1	1.5	1	1.8	0	0.0	4	0.9
Pain/ Discomfort	No	40	76.9	46	68.7	49	64.5	50	65.8	32	47.8	22	40.0	19	57.6	258	60.6
	Slight	10	19.2	17	25.4	20	26.3	10	13.2	18	26.9	22	40.0	9	27.3	106	24.9
	Moderate	2	3.8	4	6.0	7	9.2	12	15.8	13	19.4	7	12.7	5	15.2	50	11.7
	Severe	0	0.0	0	0.0	0	0.0	2	2.6	4	6.0	3	5.5	0	0.0	9	2.1
	Extreme	0	0.0	0	0.0	0	0.0	2	2.6	0	0.0	1	1.8	0	0.0	3	0.7
Anxiety/ depression	No	42	80.8	52	77.6	65	85.5	57	75.0	52	77.6	47	85.5	28	84.8	343	80.5
	Slight	5	9.6	10	14.9	7	9.2	10	13.2	12	17.9	4	7.3	2	6.1	50	11.7
	Moderate	4	7.7	3	4.5	4	5.3	7	9.2	2	3.0	4	7.3	3	9.1	27	6.3
	Severe	1	1.9	2	3.0	0	0.0	2	2.6	1	1.5	0	0.0	0	0.0	6	1.4
	Extreme	0	0.0	0	0.0	0	0.0	0	0.0	0	0.0	0	0.0	0	0.0	0	0.0
	Self- reported EQ-5D-5L raw numbers, percentages by age group: Females																
Parameter		Age														Total	
		18–24		25–34		35–44		45–54		55–64		65–74		75+			
		n	%	n	%	n	%	n	%	n	%	n	%	n	%	n	%
Total	N	36		98		145		153		115		101		57		705	
Mobility	No Problems	31	86.1	94	95.9	135	93.1	119	77.8	85	73.9	62	61.4	25	43.9	551	78.2
	Slight problems	5	13.9	3	3.1	6	4.1	21	13.7	18	15.7	18	17.8	16	28.1	87	12.3
	Moderate problems	0	0.0	0	0.0	3	2.1	12	7.8	8	7.0	17	16.8	12	21.1	52	7.4
	Severe problems	0	0.0	1	1.0	1	0.7	1	0.7	3	2.6	4	4.0	4	7.0	14	2.0
	Unable	0	0.0	0	0.0	0	0.0	0	0.0	1	0.9	0	0.0	0	0.0	1	0.1
Self-care	No Problems	36	100.0	96	98.0	141	97.2	149	97.4	102	88.7	96	95.0	39	68.4	659	93.5
	Slight problems	0	0.0	1	1.0	4	2.8	2	1.3	10	8.7	3	3.0	16	28.1	36	5.1
	Moderate problems	0	0.0	1	1.0	0	0.0	2	1.3	2	1.7	1	1.0	1	1.8	7	1.0
	Severe problems	0	0.0	0	0.0	0	0.0	0	0.0	1	0.9	1	1.0	1	1.8	3	0.4
	Unable	0	0.0	0	0.0	0	0	0	0.0	0	0.0	0	0.0	0	0.0	0	0.0
Usual activities	No Problems	31	86.1	92	93.9	131	90.3	120	78.4	88	76.5	77	76.2	29	50.9	568	80.6
	Slight problems	4	11.1	3	3.1	10	6.9	23	15.0	17	14.8	8	7.9	10	17.5	75	10.6
	Moderate problems	1	2.8	2	2.0	4	2.8	8	5.2	6	5.2	13	12.9	11	19.3	45	6.4
	Severe problems	0	0.0	1	1.0	0	0.0	1	0.7	4	3.5	2	2.0	6	10.5	14	2.0
	Unable	0	0.0	0	0.0	0	0.0	1	0.7	0	0.0	1	1.0	1	1.8	3	0.4
Pain/ Discomfort	No	27	75.0	77	78.6	103	71.0	80	52.3	58	50.4	46	45.5	24	42.1	415	58.9
	Slight	7	19.4	19	19.4	27	18.6	41	26.8	34	29.6	23	22.8	13	22.8	164	23.3
	Moderate	2	5.6	2	2.0	14	9.7	26	17.0	17	14.8	27	26.7	14	24.6	102	14.5
	Severe	0	0.0	0	0.0	1	0.7	6	3.9	6	5.2	5	5.0	4	7.0	22	3.1
	Extreme	0	0.0	0	0.0	0	0.0	0	0.0	0	0.0	0	0.0	2	3.5	2	0.3
Anxiety/ depression	No	28	77.8	76	77.6	106	73.1	113	73.9	89	77.4	83	82.2	44	77.2	539	76.5
	Slight	4	11.1	13	13.3	25	17.2	25	16.3	18	15.7	12	11.9	9	15.8	106	15.0
	Moderate	4	11.1	7	7.1	12	8.3	13	8.5	7	6.1	5	5.0	4	7.0	52	7.4
	Severe	0	0.0	1	1.0	2	1.4	2	1.3	0	0.0	1	1.0	0	0.0	6	0.9
	Extreme	0	0.0	1	1.0	0	0.0	0	0.0	1	0.9	0	0.0	0	0.0	2	0.3

**Figure 1.**
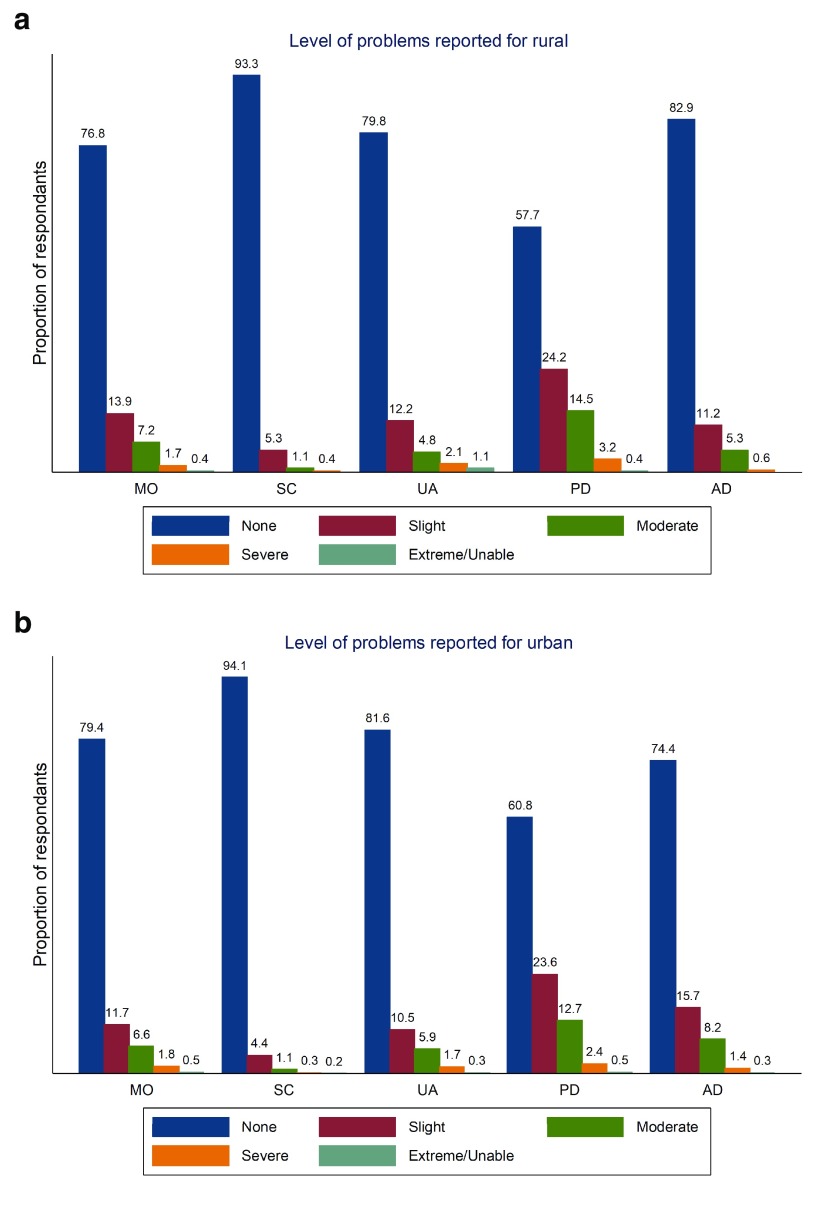
Proportion reporting problems in each EQ-5D-5L domain from rural ( **a**) and urban ( **b**).

**Figure 2.**
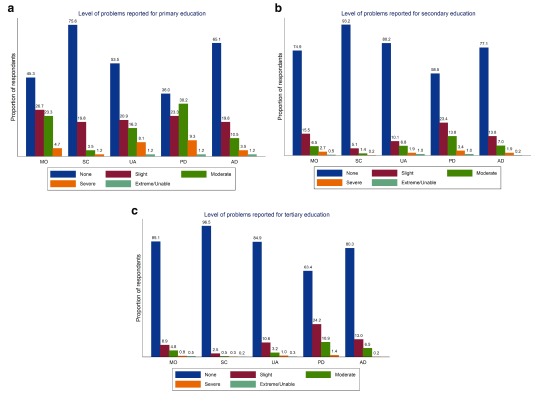
Proportion reporting problems in each EQ-5D-5L domain by primary ( **a**), secondary ( **b**), and tertiary ( **c**) education Level.

**Figure 3.**
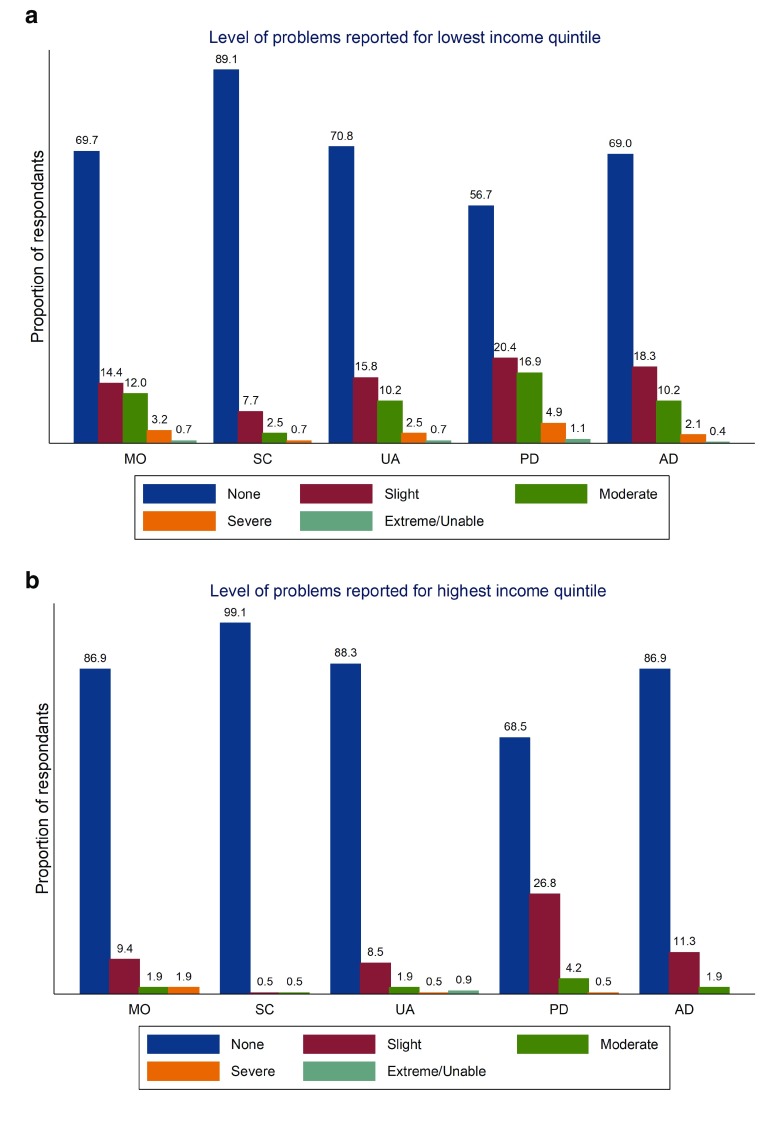
Proportion reporting problems in each EQ-5D-5L domain for lowest ( **a**) and highest ( **b**) income quintile.

**Figure 4.**
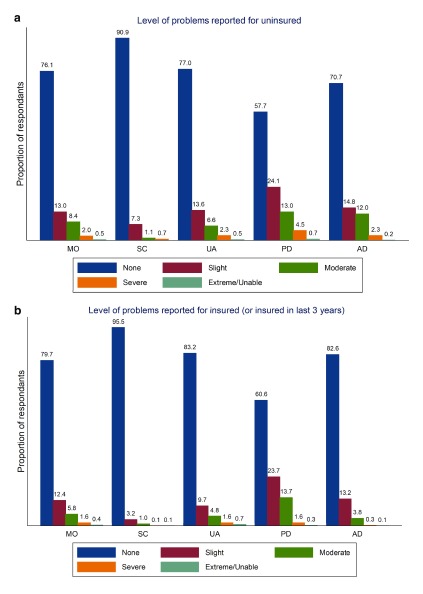
Proportion reporting problems in each EQ-5D-5L domain by uninsured ( **a**) and insurance ( **b**) status.

We estimated the percentage by urban/rural dwelling status at each level of each domain ( Figures 1a and 1b) as well by education level ( Figures 2a, 2b and 2c). In addition we estimated the percentage at each level of each domain for the highest and lowest income quintiles ( Figure 3a and 3b) and by PHI ( Figures 4a and 4b). We estimated the summary health score (as calculated above) for the entire sample as well as by age and gender presenting these as means with standard deviations and medians with interquartile ranges repeating this in respect of the visual analogue scale scores for comparative purposes ( Table 4).

**Table 4.** Mean and median VAS and sum score in total and by age group and gender along with standard deviation and inter quartile ranges.

	VAS					Sum score					
	Mean	SD	Median	Lower IQR	Upper IQR	Mean	SD	Median	Lower IQR	Upper IQR	N
Total	79.9	14.9	80	70	90	91.8	11.9	95	90	100	1,131
Female	80.2	15.3	80	70	90	91.5	12.3	95	90	100	705
Male	79.5	14.2	80	70	90	92.2	11.2	95	90	100	426
18–24	80.7	14.9	82.5	72.5	90	95.6	6.3	100	95	100	88
25–34	83.0	13.2	87	75	90	95.9	6.9	100	95	100	165
35–44	81.6	13.1	82	75	90	95.0	8.2	100	90	100	221
45–54	79.3	16.5	80	70	90	91.4	11.8	95	85	100	229
55–64	79.3	14.3	80	70	90	89.9	13.4	95	90	100	182
65–74	78.6	16.0	80	70	90	87.9	14.6	95	80	100	156
75+	74.9	15.6	80	66	90	84.1	15.4	90	75	100	90

IQR – Inter Quartile Range, VAS – Visual Analogue Scale, SD – Standard Deviation

We examined the relationship between the summary score and the characteristics of the respondent in a generalised linear regression model using a Poisson family and power link of 0.6 where age, gender, education, income, rural/urban dwelling status and private health insurance status were regressed on the summary score ( Table 5). This analysis was repeated where the sample was weighted for age and gender to more closely reflect that of the population at large; results are reported in Supplementary File 2. We re-estimated this model separately with respect to each domain using an ordered logit model. Marginal effects for this were computed and presented for income, private insurance and education ( Table 6a, Table 6b and Table 6c). Finally, as part of an examination of the distribution of health across the sample we estimated a concentration curve and concentration indices for the summary score and each of the individual domains in which equivalised income served as the ranking variable, the result is reported in Supplementary File 3. Equivalised income was calculated as household income divided by the square root of the household number ^36^. All analyses were conducted using STATA version 14.0.

**Table 5.** Average marginal reduction in sumscore associated with a unit change in each covariate. Results from a generalised linear model with a Poisson family and power link of 0.6. For example every year of age reduces sumscore by 0.17 units while being in tertiary education increases sumscore by 5.21 units *ceteris paribus*.

	dy/dx	95% Confidence Interval (CI)	
		Lower CI	Upper CI
Age	0.17	0.13	0.21
Male	0.11	-1.13	1.35
Ed. Level (Base: Primary)			
Secondary	-4.56	-7.75	-1.36
Tertiary	-5.21	-8.47	-1.94
Income Quintile (Base: lowest)			
2	-2.12	-4.19	-0.05
3	-1.94	-3.97	0.09
4	-3.07	-5.10	-1.04
5	-4.11	-6.18	-2.04
Urban	0.40	-0.82	1.62
Private Insurance (or last 3 years)	-1.51	-2.95	-0.06

**Table 6a.** Marginal effects of income.

Base: Income quintile 1 (lowest)		Mobility	Self-care	Usual Activities	Pain/ Discomfort	Anxiety/ Depression
Income 2	No problems	0.0747 ***	0.0132	0.0601 **	0.0307	0.0541 *
	Slight problems	-0.0459 ***	-0.0103	-0.0352 **	-0.0145	-0.0325
	Moderate problems	-0.0222 ***	-0.0021	-0.0174 **	-0.0129	-0.0183 *
	Severe problems	-0.0053 **	-0.0007	-0.0057 **	-0.0028	-0.0028
	Extreme/Unable	-0.0013 *	-0.0002	-0.0019 *	-0.0005	-0.0005
Income 3	No problems	0.0579 **	0.0061	0.0806 ***	-0.0059	0.0069
	Slight problems	-0.0354 **	-0.0048	-0.0473 ***	0.0027	-0.0041
	Moderate problems	-0.0174 **	-0.0009	-0.0232 ***	0.0026	-0.0024
	Severe problems	-0.0041 **	-0.0003	-0.0076 ***	0.0006	-0.0003
	Extreme/Unable	-0.001	-0.0001	-0.0025 **	0.0001	-0.0001
Income 4	No problems	0.0712 **	0.0204 *	0.0926 ***	0.0494	0.0626 *
	Slight problems	-0.0437 **	-0.0159 *	-0.0544 ***	-0.0235	-0.0376 *
	Moderate problems	-0.0213 **	-0.0032 *	-0.0266 ***	-0.0207	-0.0212 *
	Severe problems	-0.0051 **	-0.001	-0.0087 ***	-0.0045	-0.0033 *
	Extreme/Unable	-0.0012 *	-0.0003	-0.0028 **	-0.0007	-0.0005
Income 5	No problems	0.0789 **	0.0376 ***	0.1007 ***	0.1177 **	0.1203 ***
	Slight problems	-0.0485 **	-0.0294 ***	-0.0594 ***	-0.0584 **	-0.0733 ***
	Moderate problems	-0.0235 **	-0.0058 **	-0.0289 ***	-0.0477 **	-0.0399 ***
	Severe problems	-0.0056 **	-0.0019 *	-0.0094 ***	-0.01 **	-0.0061 ***
	Extreme/Unable	-0.0014 *	-0.0005	-0.0031 **	-0.0016 *	-0.001

Marginal effects are presented for just income though regressions are also controlled for urban/rural status, age group (18–35, 34–45, 44–60, 61+), sex, secondary education (Y/N), third level education (Y/N) and private insurance (or in the last 3 years: Y/N) *** significant at 0.01, ** significant at 0.05, * significant at 0.1

**Table 6b.** Marginal effects of private insurance.

Base: No Insurance		Mobility	Self-care	Usual Activities	Pain/Discomfort	Anxiety/Depression
Private Insurance (or in the last 3 years)	No problems	0.0086	0.0169	0.0144	0.0174	0.0719 **
	Slight problems	-0.0052	-0.0132	-0.0083	-0.0079	-0.0419 **
	Moderate problems	-0.0026	-0.0027	-0.0043	-0.0075	-0.0254 **
	Severe problems	-0.0006	-0.0009	-0.0014	-0.0017	-0.0039 **
	Extreme/Unable	-0.0002	-0.0002	-0.0005	-0.0003	-0.0007

Marginal effects are presented for just Insurance though regressions are also controlled for urban/rural status, age group (18–35, 34–45, 44–60, 61+), sex, secondary education (Y/N), third level education (Y/N) and income quintiles (base: lowest) *** significant at 0.01, ** significant at 0.05, * significant at 0.1

**Table 6c.** Marginal effects of education.

Base: Primary Education		Mobility	Self-care	Usual Activities	Pain/Discomfort	Anxiety/Depression
Secondary Education	No problems	0.0788 **	0.0229 **	0.1041 ***	0.1644 ***	0.1132 ***
	Slight problems	-0.0477 **	-0.0179 **	-0.0602 ***	-0.0785 ***	-0.0673 ***
	Moderate problems	-0.0239 **	-0.0036 *	-0.0305 ***	-0.0688 ***	-0.0389 ***
	Severe problems	-0.0057 **	-0.0012	-0.01 ***	-0.0148 ***	-0.006 **
	Extreme/Unable	-0.0014 *	-0.0003	-0.0033 **	-0.0024 *	-0.001
Tertiary Education	No problems	0.1117 ***	0.0244	0.1043 **	0.1649 ***	0.132 ***
	Slight problems	-0.0662 ***	-0.019	-0.059 **	-0.0729 ***	-0.0763 ***
	Moderate problems	-0.0349 **	-0.0038	-0.0313 **	-0.0729 ***	-0.047 **
	Severe problems	-0.0085 **	-0.0013	-0.0105 **	-0.0164 **	-0.0075 **
	Extreme/Unable	-0.0021 *	-0.0003	-0.0035 **	-0.0027 *	-0.0012

Marginal effects are presented for just Education though regressions are also controlled for urban/rural status, age group (18–35, 34–45, 44–60, 61+), sex, private insurance (or in the last 3 years: Y/N) and income quintiles (base: lowest) *** significant at 0.01, ** significant at 0.05, * significant at 0.1

## Results

Table 1 to Table 6 and Figure 1 to Figure 6 present the results as described above. As can be seen from Table 1 the sample is broadly representative of the population albeit with over-representation of older age groups and of females. In Table 2 and Table 3 we see that those who are older and females tend to report poorer health than those who are younger and males. Interestingly the pattern of difference is not uniform across domains of health. Males and females, for example, are more alike with respect to mobility, self-care and usual activities and more divergent with respect to pain/discomfort and anxiety/depression. Similarly, with respect to age, while there is an evident deterioration in health across domains associated with ageing, this is more marked in domains such as mobility, usual activities and pain/discomfort compared with anxiety/depression and self-care.

As seen in Figure 1 those who are urban dwellers generally experience better health compared to rural dwellers with the exception of anxiety/depression where urban dwellers exhibit poorer health. With regard to socio-economic characteristics those who are better educated report better health across all domains than those who are less well educated ( Figure 2) though again there are evident differences between domains – differences with anxiety/depression being less evident than in other domains. Similarly, those with higher incomes ( Figure 3) report better health across all domains though again differences are less evident across some domains. In Figure 4 it is seen that those with PHI enjoy better health than those without PHI. As with other analyses the degree of divergence between sub-groups differentiated by PHI status is seen to vary across domains, differences in anxiety/depression for example being less evident than with respect to the other domains.

**Figure 5.**
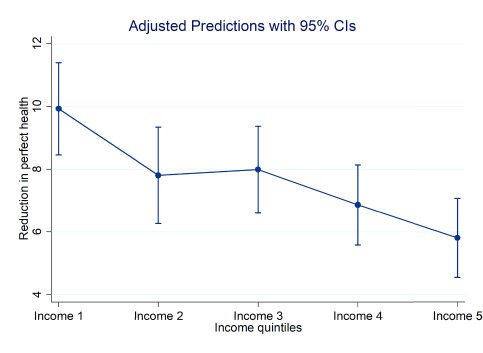
Average marginal reduction in sumscore with 95% confidence intervals for each income quintile holding covariates at their mean value.

**Figure 6.**
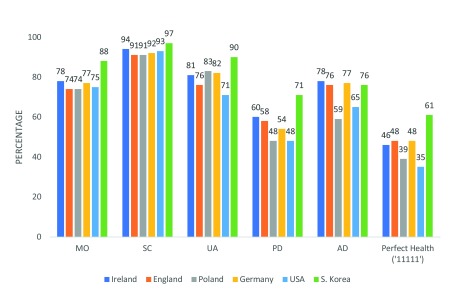
Prevalence of ‘No problems’ reported using EQ-5D-5L.

In Table 4 and consistent with the results reported in Table 2 and Table 3 we see the mean crude summary score for those who are older and those who are female are lower than for those who are younger and those who are male respectively – though in the case of gender the difference is small and reversed with respect to the visual analogue scale. In Table 5 we see that controlling for the covariates identified, age remains a significant determinant of self-report health. Based on an examination of z-tests, we see that socio-economic characteristics are significant determinants of health measured using the crude summary score. This pattern is repeated with respect to individual domains as seen in Table 6. With respect to income, for example, we see that those with higher incomes are more likely to report no problems and less likely to report severe problems. Comparing mobility and anxiety/depression, however, we see that relative to the lowest income quintile those in the highest income quintile are 7.9 percentage points more likely to report no problems in mobility and 0.14 percentage points less likely to report being extreme/unable in this domain. By comparison with respect to anxiety/depression those in the highest income quintile are almost 12 percentage points more likely to report no problems and 0.1 percentage points less likely to be extreme/unable in this domain. Thus, the degree of difference between socio-economic groups does vary across domains. For illustrative purposes in Figure 5 the marginal effects with respect to income together with their confidence intervals are shown. The negative coefficient and high degree of statistical significance for the concentration indices in Supplementary File 3 shows - consistent with the results in Table 5 and Table 6 - that poor health is disproportionately experienced by those on lower incomes. In Figure 6 Ireland’s norms across domains are presented alongside those of a number of other countries. In Table 7 the most frequently reported health states among the population are reported. Interestingly the top 6 are ranked in the same order as in England and 8 out of the top 10 are the same health states in Ireland as in the England study ^17^.

**Table 7.** Most frequently reported EQ-5D-5L.

Health State	n	%	Cum. %
11111	516	45.62	45.62
11121	119	10.52	56.15
11112	66	5.84	61.98
11131	36	3.18	65.16
11122	27	2.39	67.55
21121	27	2.39	69.94
11221	21	1.86	71.79
11113	20	1.77	73.56
21111	19	1.68	75.24
21131	11	0.97	76.22
11123	10	0.88	77.10
21221	9	0.80	77.90
21231	9	0.80	78.69
11231	8	0.71	79.40
21211	7	0.62	80.02
31131	7	0.62	80.64
11133	6	0.53	81.17

Health State represents response for each dimension (Mobility, self-care, Usual activities, pain/discomfort, anxiety/depression) 1=no problem, 2 = slight problem 3 = moderate problem of the 5 levels e.g. 31131 = moderate problems in mobility, no problems in self-care, no problems in usual activities, moderate pain/discomfort, not anxious or depressed.

## Discussion

Our findings provide the first description of population health for Ireland using the EQ-5D-5L descriptive system. Consistent with other international studies using the 5L system they reveal a pattern in which health appears to be better among males than females (albeit marginally) and among those who are younger relative to those who are older ^17, 29– 33^. Distinct patterns are evident with respect to age and gender across domains – patterns not dissimilar to those observed elsewhere. While the percentage of females reporting no difficulties with respect to anxiety/depression and pain/discomfort, for example, were in England 73.9% and 55.7% the respective figures for males were 79% and 62.5% ^17^. In Ireland, while the percentage of females reporting no difficulties in respect of anxiety/depression and pain/discomfort were 76.5% and 58.9% the respective figures for men were 80.5% and 60.6%. Why females should self-report poorer health may relate to differential *exposure* to health risks, (arising, for example, through employment in more stressful jobs or workplaces that are more stressful for women; greater stress/physical effort associated with the provision of informal care or the work and responsibilities associated with pregnancy). Equally, females may experience greater *vulnerability* to health risks (arising for example as a result of lower access to resources such as higher incomes through which the effects of ill-health can be ameliorated). It is also possible that exposure and vulnerability may combine to contribute to differences as is the possibility that there is differential reporting bias associated with different mapping structures between objective and subjective health for males and females ^37, 38^. We though, have no evidence to either support or refute this argument.

That differences between genders differ across domains of health such as usual activities, self-care and mobility is consistent with intuition and echoes findings from England. However, that gender is not significant in our regression analyses when socio-economic characteristics are controlled for is noteworthy. This could be construed as supporting the argument that differences between males and females are grounded in issues of exposure and vulnerability as suggested above, though further research effort could usefully be devoted to this issue.

More broadly, based on the percentage of those reporting no problems in respect of the various domains, the health of those resident in Ireland appears to be similar to that in England and Germany and slightly better than those in Poland or the USA. As with the other studies and with the exception of anxiety/depression, population self-reported health appears to be broadly poorer than in South Korea. Differences between countries, however, are apparent with respect to specific domains of health. While, for example, the percentage of those with no problems in respect of mobility, was in the mid to high 70s in England, Poland, Ireland, Germany and the USA, in the low 90s in respect of self-care and mid-70s to low 80s in respect of usual activities for England, Poland, Ireland and Germany, with respect to pain/discomfort and anxiety/depression greater heterogeneity is apparent ( Figure 6). With respect to pain/discomfort and anxiety/depression, Ireland, England and Germany exhibited similar health to each other and better health than Poland and the USA. While almost 60% in Ireland and England and 54.4% in Germany reported no problems with respect to pain/discomfort, in Poland and the USA the figures were roughly 48% (in South Korea it was 71%). Similarly, with respect to anxiety/depression while in Ireland, England and Germany approximately 77% reported no problems, in Poland and the USA the respective figures were roughly 59% and 65% (in South Korea roughly 76%). The potential for cultural factors to explain differences in health has been mooted ^39^ and might be interpreted in a similar fashion to reporting bias. The potential for differences in exposure and in vulnerability to health risks though may also provide explanations for the observed differences. The differences reported here, if replicated in other countries, however, also suggest possibly greater similarities within Western Europe compared with the USA and Eastern Europe. Given the small number of countries available this must be viewed cautiously but is an issue worthy of further investigation.

Within Ireland the gradient in health related to age, as noted, is consistent with that found elsewhere and is consistent with intuition. That physical aspects of health appear to be more directly correlated with age than, for example, anxiety/depression, is perhaps to be expected, similar trends being evident elsewhere albeit being more marked in Ireland and England than with Poland. For example, the percentage reporting no problems in anxiety/depression in England rose from 73% among those aged 65 to over to 83% among those under 35. In Ireland older persons actually exhibited slightly better mental health among the over 65s with 82% reporting no problems compared to 78% among the under 35s. In Poland by contrast among those aged under 35 approximately 75% reported no problems but only just over 40% of those over 65 reported no problems. It is unclear what factors may lie behind these differences; it could for example relate to the relatively high levels of migration by young persons from Poland or the legacy effects of previous economic hardship. The greater similarity between Ireland and England compared to Poland is in any event evident. Similarly, with respect to mobility the percentage with no problems rose from 50% among those aged over 65 to 92% in those aged under 35 in England, while in Ireland the comparable figures were 53% and 94%. By contrast in Poland the percentage with no problems among the under 35 was approximately 95% and among those over 65 it was approximately 38%, again evidencing a sharper decline; this is similarly noted in respect of pain and discomfort. Over-interpretation of the data is dangerous but that healthy ageing may not be experienced to the same degree across countries is certainly a possible explanation.

With respect to socio-economic characteristics within Ireland there is clear evidence of a socio-economic gradient in health with respect to income, education and PHI status. Those who are better educated, who have higher incomes and who have PHI are seen to have better self-reported health with respect to the crude summary score and across domains than those who are less well educated, have lower incomes and who don’t have PHI. That socio-economic differences appear to be sharper when measured with respect to education than income is evident though some caution is warranted in the interpretation of these results. While both education and income relate to socio-economic status, in Ireland there also exists a correlation between age and education that may confound results. Those over 65 are more likely than younger age cohorts to have been educated only to primary level. This may in part explain the differences evident in univariate comparisons with respect to education. This may also explain the sharper differences with respect to aspects of physical health when using education alone to compare differences in socio-economic status than income. In Figure 2 for example, while there are almost 40 percentage points between those with the highest and lowest levels of education with respect to no problems in mobility (45.3% versus 85.1%) there is approximately only a 15 percentage point difference between these groups with respect to anxiety/depression (80.3% versus 65.1%). By contrast in Figure 3 we see that the differences between the highest and lowest income quintiles are approximately 17 (86.9% and 69.7%) and 18 percentage points (86.9% and 69%) for mobility and anxiety/depression respectively. By contrast in regression analyses where covariates – notably age – are controlled for a sharper socio-economic gradient with respect to income is evident in respect of anxiety/depression than with respect to other domains. In Table 6, for example, we see that there is just over a 12 percentage point difference between the highest and lowest income quintiles in the probability of reporting no problems compared to a roughly 8 percentage point difference in respect of mobility. Similarly, with respect to the concentration indices, a shaper gradient in respect of anxiety/depression is evident than with respect to mobility. Again the relationship between specific indicators of socio-economic status and specific domains of health is an area where further research effort could usefully be expended. 

Focusing on income as an indicator of socio-economic status it is clear from the ordered logits that there is a protective effect related to income and that the protection offered by higher income varies between domains. That income offers no statistically significant benefit in terms of the likelihood of being in the lowest category of health in each domain (usual activities being the exception) is likely to be a function of the relatively small numbers reporting difficulties at the more extreme levels in the survey. With respect to higher levels of health, it is evident that income is significantly related to reporting no problems in each domain, a clear dose response also being evident – those in the highest income quintile enjoying the greatest increase in probability of being in the no-problems groups. The patterns may indicate that while those who are better off are in general healthier than those who are less well off, when it comes to extreme levels of poor health a socio-economic gradient is less likely to be evident – the socio-economic gradient narrowing at extreme levels of poor health. Whether these differences reflect differences in vulnerability, exposure or reporting bias is unclear. The relationship between ageing, health, and healthcare expenditures has been the subject of considerable research effort ^40– 42^. Our findings may help encourage further examination of socio-economic gradients in these relationships investigating, for example, whether they are stable across socio-economic groups and/or types of service used. Similarly as descriptive studies of this type are repeated over time they may help shed light on not just how healthily different parts of the population are ageing but what impact policy, life style and economic factors have on this.

With respect to health insurance, in univariate analysis that those with PHI report better health in general (the crude summary score) and across each domain is clear. It is probable that this is in part explained by PHI being positively associated with income and education, both of which may affect the opportunity and ability of respondents to invest in their health (i.e. reduce their vulnerability) consistent with the Grossman model ^43^ and/or to mitigate their exposure to health risks. There is for example, a literature pointing to a greater use of tertiary services by those with private insurance in Ireland ^44^ – services one would imagine improve health. The difference in health between those with and without insurance does not attain statistical significance – with the exception of anxiety/depression – when other socio-economic characteristics are controlled for as seen in Table 6, is noteworthy. This may support the argument that health differences are related to socio-economic status rather than insurance per se. While insurance status remains significant with respect to anxiety/depression this may reflect a greater sense of security conferred by having insurance or associated with insurance status through some other unobserved variable. That those with PHI may attach different values to health has also been mooted in the literature ^44, 45^. If this were the case, those with PHI might be reasonably expected to engage in other health seeking behaviours that help to preserve health relative to those without PHI. By extension that there may also be differences with respect to specific domains of health is conceivable. Whether those with PHI attach different values to health is an issue on which further research is required. 

There are a two main limitations to our study. First, we concede that our sample does contain some over-representation, despite purposive sampling undertaken to boost under-represented groups. This said, it is large, broadly representative and analyses based on weighted data found no material differences in results. While we could have adopted an approach used in some other studies of constructing a sample based on quotas – for example by age and sex ^21^ - a challenge with this approach is in ensuring representation across multiple socio-demographic characteristics. As noted by Szende *et al*. ^46^, moreover “Because the population norms are presented by age and gender, there is no need for the sample to have the same age distribution as the general population…” when making comparisons. On balance we think having a richer characterization of the sample in terms of their socio-demographic characteristics was worthwhile; researchers can if they wish re-weight the data as they deem appropriate.

Second, we did not collect EQ-5D-3L data, data on health care use or data on doctor diagnosed conditions among the information we collected. In each instance a case could be readily made for the usefulness of these additional data for example to contextualise the other information collected. Given the risks respondent fatigue posed to the quality of the data actually collected, however, a balance must be struck. We chose to safeguard the quality of the preference and health status data rather than potentially jeopardise this by collecting interesting but arguably unnecessary information. Given the aims of our research we think this decision is justified.

## Conclusions

As part of a larger study that examined preferences for health states in Ireland ^47, 48^ we have presented population norms of self-reported health based on the EQ-5D-5L descriptive system for Ireland. They demonstrate that those resident in Ireland reported being in relatively good health compared to other similar countries where EQ-5D-5L data have been collected such as England. Among residents of Ireland, self-reported health was higher among those who were younger compared to those who were older; among those who were better educated compared to those less well educated; among those who had higher incomes compared to those with lower incomes; among those who had private health insurance compared to those who had not and; among those who lived in urban areas compared to those who lived in rural areas. While men had slightly better health than women, the differences were small. Heterogeneity in health was evident across domains, in a manner that could be explained by reference to differences in vulnerability and exposure to health risks and different socio-economic gradients across domains worthy of further investigation were evident. These norms will be of use to those collecting EQ-5D-5L data wishing to compare their study samples against those of the population at large. They provide baseline data against which the health of the population can be measured in the future as demographic and economic conditions change. The data provides a resource to those interested in examining self-reported health related quality of life in Ireland or in comparing health in Ireland with that in other countries. 

## Data availability

All data used in these analyses are freely available from the Irish Social Science Data Archive from following the link:

http://www.ucd.ie/issda/data/irisheq-5d-5lsurvey2015-2016/

### Accessing the data

To access the data, please complete a ISSDA Data Request Form for Research Purposes, sign it, and send it to ISSDA by email ( issda@ucd.ie).

For teaching purposes, please complete the ISSDA Data Request Form for Teaching Purposes, and follow the procedures, as above. Teaching requests are approved on a once-off module/workshop basis. Subsequent occurrences of the module/workshop require a new teaching request form.

Data will be disseminated on receipt of a fully completed, signed form.

## Supplementary material

Supplementary File 1 - Socio-demographic questionnaire.

Supplementary File 2 - Weighted generalised linear model results for average marginal reduction in sumscore associated with a unit change in each covariate.

Supplementary File 3 – EQ-5D-5L Population Norms: Concentration indices and curves.
