# Supplementary material for: The health of the residents of Ireland: Population norms for Ireland based on the EQ-5D-5L descriptive system – a cross sectional study
Source: HRB Open Res. 2018 Sep 4;1:22. [Version 1] doi: 10.12688/hrbopenres.12848.1 (PMC6973536; doi:10.12688/hrbopenres.12848.1)
Supplement: Supplementary file 1 [file hrbopenres-1-13910-s0000.tgz › 01319bb1-0ce1-48eb-a171-60a775f3c8cb_Questionnare_The_Irish_EQ-5D-5L_Survey__2015_-_2016.pdf]

The Irish EQ-5D-5L Survey

2015-2016

Questionnaire

Under each heading, please tick the ONE box that best describes your health TODAY.

MOBILITY

I have no problems in walking about ❑

I have slight problems in walking about ❑

I have moderate problems in walking about ❑

I have severe problems in walking about ❑

I am unable to walk about ❑

SELF-CARE

I have no problems washing or dressing myself ❑

I have slight problems washing or dressing myself ❑

I have moderate problems washing or dressing myself ❑

I have severe problems washing or dressing myself ❑

I am unable to wash or dress myself ❑

USUAL ACTIVITIES *(e.g. work, study, housework, family or leisure activities)*

I have no problems doing my usual activities ❑

I have slight problems doing my usual activities ❑

I have moderate problems doing my usual activities ❑

I have severe problems doing my usual activities ❑

I am unable to do my usual activities ❑

PAIN / DISCOMFORT

I have no pain or discomfort ❑

I have slight pain or discomfort ❑

I have moderate pain or discomfort ❑

I have severe pain or discomfort ❑

I have extreme pain or discomfort ❑

ANXIETY / DEPRESSION

I am not anxious or depressed ❑

I am slightly anxious or depressed ❑

I am moderately anxious or depressed ❑

I am severely anxious or depressed ❑

I am extremely anxious or depressed ❑

sample

Source:https://euroqol.org/wp-content/uploads/2016/10/Sample_UK__English__EQ-5D-5L_Paper_Self_complete_v1.0__ID_24700.pdf

- We would like to know how good or bad your health is TODAY.
- This scale is numbered from 0 to 100.
- 100 means the <u>best</u> health you can imagine.
  0 means the <u>worst</u> health you can imagine.
- Mark an X on the scale to indicate how your health is TODAY.
- Now, please write the number you marked on the scale in the box below.

YOUR HEALTH TODAY = ☐

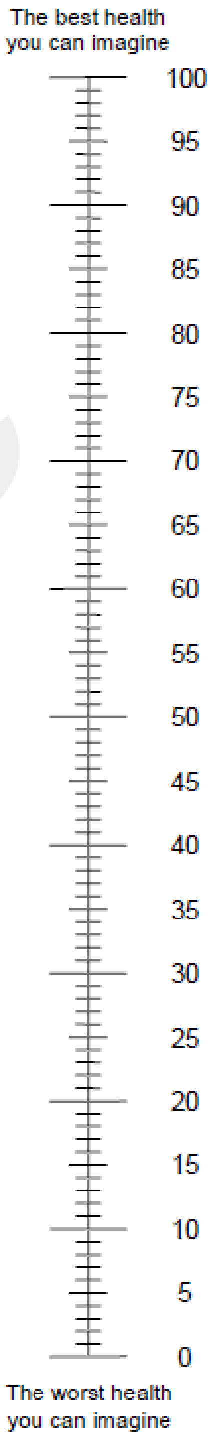

Sample

Source:https://euroqol.org/wp-content/uploads/2016/10/Sample_UK__English__EQ-5D-5L_Paper_Self_complete_v1.0__ID_24700.pdf

Screen Shots from EuroQol Valuation Technology (EQ-VT Software).

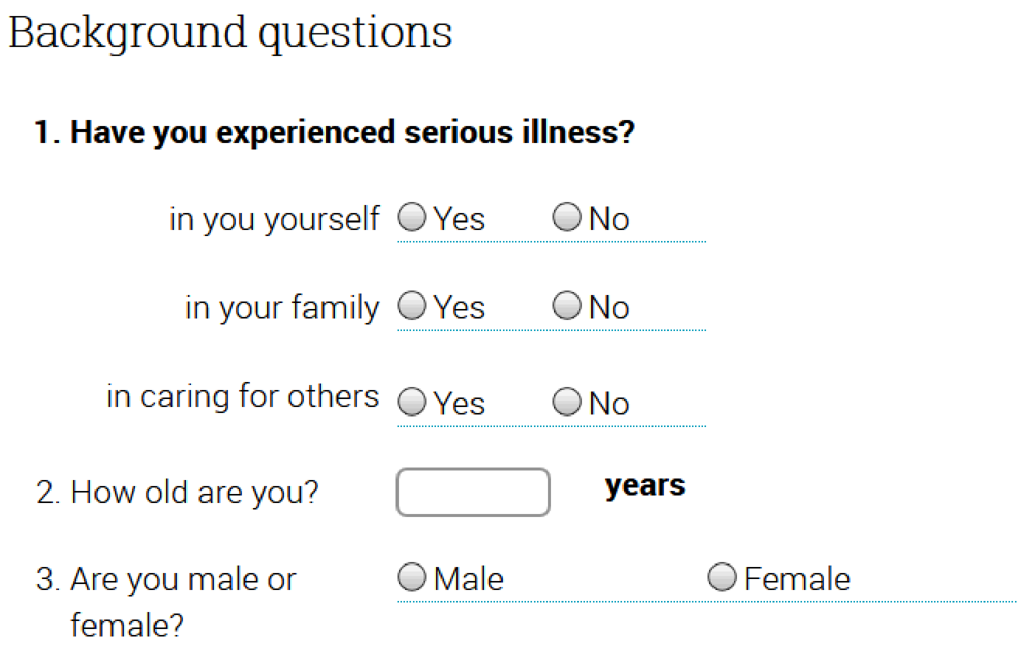

Background questions

1. Have you experienced serious illness?

in you yourself ◯ Yes ◯ No

in your family ◯ Yes ◯ No

in caring for others ◯ Yes ◯ No

2. How old are you? [ ] **years**

3. Are you male or female? ◯ Male ◯ Female

Irish Local Questionnaire

1. Do you have private medical insurance?

- ◯ Yes
- ◯ No

2. Have you had private medical insurance in the last 3 years?

- ◯ Yes
- ◯ No

3. Do you have a medical card?

- ◯ Yes, full medical card
- ◯ Yes, GP visit card
- ◯ Neither

4. Which of these best describes your usual situation in regard to work?

- ◯ Full time Employed/Self Employed
- ◯ Part time Employed/Self Employed
- ◯ Unemployed
- ◯ Student
- ◯ Long-term sickness or disability
- ◯ Home duties/looking after home or family
- ◯ Retired
- ◯ Other (specify)

5. What is the highest level of education you have completed to date?

- ◯ Primary or less
- ◯ Second Level or less
- ◯ Third Level

6. How many years of full time education have you completed?

- ◯ 8 years or less
- ◯ 9 to 13 years
- ◯ More than 13 years

7. What is you ethnic or cultural background?

- ◯ Irish
- ◯ European (non- Irish)
- ◯ Other (specify)

8. Would you describe the place where your household is situated as being...?

- ◯ Urban
- ◯ Rural

9. What is the total of your annual household income?

- ◯ €0 - €10,000
- ◯ €10,001 - €20,000
- ◯ €20,001 - €30,000
- ◯ €30,001 - €40,000
- ◯ €40,001 - €50,000
- ◯ €50,001 - €60,000
- ◯ €60,001 - €75,000
- ◯ €75,001 - €100,000
- ◯ €100,001 - €200,000
- ◯ > €200,001

10. How many people in total (including yourself and all children) live regularly as members of your household?

[ ]

11. Of these how many are children under 18 years?

[ ]

12. What is your current marital status?

- ◯ Married/Living as married
- ◯ Never Married
- ◯ Divorced/Separated
- ◯ Widowed

13. Do you regard yourself as belonging to any particular religion?

◯ Yes ◯ No

14. Which of these religions do you regard yourself as belonging to?

- ◯ Roman Catholic
- ◯ Other Christian
- ◯ Hindu
- ◯ Jewish
- ◯ Muslim
- ◯ Other Please specify

15. Apart from such special occasions as weddings and funerals, how often nowadays do you attend services or meetings connected with your religion?

- ◯ At least once a week
- ◯ At least once a month
- ◯ A few times a year
- ◯ Never or practically never

Note: *On occasion data was trimmed or categorised to preserve anonymity*

Any queries please contact:

Professor Ciaran O'Neill,

Queen's University Belfast, Northern Ireland.

Ciaran.oneill@qub.ac.uk
