# Supplementary material for: The health of the residents of Ireland: Population norms for Ireland based on the EQ-5D-5L descriptive system – a cross sectional study
Source: HRB Open Res. 2018 Sep 4;1:22. [Version 1] doi: 10.12688/hrbopenres.12848.1 (PMC6973536; doi:10.12688/hrbopenres.12848.1)
Supplement: Supplementary file 2 [file hrbopenres-1-13910-s0002.tgz › 66d7821a-5b0d-4ac4-9f85-59efd1ddef26_Supplementary_File_2.docx]

# Supplementary File 2

Weighted generalised linear model results for average marginal reduction in sumscore associated with a unit change in each covariate. Weighted by age group and gender from CSO 2011 figures, see table 5 for unweighted results.

|  | dy/dx | 95% Confidence Interval | |
| --- | --- | --- | --- |
|  |  |  |  |
| Age | 0.16 | 0.02 | 0.30 |
|  |  |  |  |
| Male | 0.13 | -4.06 | 4.32 |
| Ed. Level (Base: Primary) |  |  |  |
| Secondary | -4.19 | -15.61 | 7.23 |
| Tertiary | -5.09 | -16.66 | 6.48 |
| Income Quintile (Base: lowest) | |  |  |
| 2 | -1.83 | -9.51 | 5.85 |
| 3 | -2.05 | -9.58 | 5.48 |
| 4 | -2.97 | -10.88 | 4.94 |
| 5 | -3.95 | -11.59 | 3.69 |
| Urban | 0.58 | -3.60 | 4.76 |
| Private Insurance (or last 3 years) | -1.12 | -6.23 | 3.98 |
