# Supplementary material for: The health of the residents of Ireland: Population norms for Ireland based on the EQ-5D-5L descriptive system – a cross sectional study
Source: HRB Open Res. 2018 Sep 4;1:22. [Version 1] doi: 10.12688/hrbopenres.12848.1 (PMC6973536; doi:10.12688/hrbopenres.12848.1)
Supplement: Supplementary file 3 [file hrbopenres-1-13910-s0001.tgz › 12f726ea-76b1-413f-830c-9b1f0f28f99e_Supplementary_File_3.docx]

# Supplementary File 3

# EQ5D5L Population Norms – Concentration indices and curves^[[1]](#endnote-1)^

## Concentration index and curve for sumscore ranked by equivalised income

### Basic, Wagstaff, Erreygers

## Concentration index and curve for sumscore ranked by alternative SES variables

### Wagstaff index recommended for bounded variables

## Concentration index and curve for Mobility ranked by equivalised income

## Concentration index and curve for Self-care ranked by equivalised income

## Concentration index and curve for Usual Activities ranked by equivalised income

## Concentration index and curve for Paid/Discomfort ranked by equivalised income

## Concentration index and curve for Anxiety/Depression ranked by equivalised income

1. Note that for the concentration curves we are looking at the distribution of ill-health so a concentration curve above the 45 degree line shows a greater concentration of ill-health in lower income groups [↑](#endnote-ref-1)
