# Supplementary material for: The health of the residents of Ireland: Population norms for Ireland based on the EQ-5D-5L descriptive system – a cross sectional study
Source: HRB Open Res. 2018 Sep 4;1:22. [Version 1] doi: 10.12688/hrbopenres.12848.1 (PMC6973536; doi:10.12688/hrbopenres.12848.1)
Supplement: Peer review history

# Peer review: Reviewer response for version 1

**DOI:** 10.21956/hrbopenres.13910.r26393

**Published:** 2018-11-06

**Authors:** Nicola McCaffrey

**Affiliations:** 1 Deakin Health Economics, Centre for Population Health Research (CPHR), School of Health and Social Development, Deakin University, Burwood, Vic, Australia

Please note, the views expressed belong solely to this reviewer. The comments are intended to be constructive and the feedback aims to improve the quality of the manuscript.

 This cross-sectional study presents new findings on the health of a representative sample (largely) of the population in Ireland. The manuscript is well-structured and very comprehensive, the methods used appear robust and the claims are generally scientifically sound, although somewhat speculative in places.

 The title adequately describes the study. The abstract is a fair summary of the study, although presenting the explicit aim(s) would be informative and including a description of which measures were used to determine socio-economic status.

 The background states the objective of the study, but again, an explicit description of how socio-economic status is being measured would be beneficial. The description of the EQ-5D-5L (paragraph two), scoring and preference weights would benefit from further editing; I found this section confusing. Perhaps provide an example health state, e.g. 21231, and how this health state would be scored to aid comprehension.

 Overall, the methods chosen appear robust and appropriate for the stated objective based on the information provided. More information is needed about the choice of regression models, including appropriate citations and the *a priori *hypotheses for the relationships should be presented. Sufficient data are provided to support the overall presentation of results. Some of the differences in health between subgroups appear small, for example the differences between domains for rural versus urban populations. How do the authors judge a meaningful difference?

 The manuscript would potentially benefit from a tighter focus on new and unexpected/ unanticipated findings, particularly in relation to the exploration of differences in health related to socio-economic status. I found the volume of results and length of the discussion somewhat overwhelming. The discussion section could be substantially reduced. For example, the results on the relationships between health status and age and gender are well-reported (as cited by the authors) and a general summary would suffice in this context. Further, there is comprehensive comparison between these results and those from other countries, yet this objective is not explicitly stated in the introduction or analysis section and comparison with other countries is missing with respect to income, education and PHI status, the more novel aspects of the analyses. Again, a brief summary of the univariate analyses would perhaps suffice with more focus placed on the regression analyses and the interpretation of the average marginal reduction (and effect sizes). Some of the discussion around the relationships between the domains and socio-demographic characteristics seems very speculative and other than pointing out further research is warranted to explore these relationships further, potentially unnecessary given the length of the manuscript. The discussion section could be further reduced by removing the specific examples and pointing readers to the relevant table or figure. Finally, the results suggest differences between the mean EQ-5D-5L VAS scores and mean sum scores and a discussion of these differences should be included in the discussion.

 The conclusion covers the main points and is a fair summary of the research.

 Generally, tables and figures are adequately presented. Maybe just present the total N and percentages in Tables 2 and 3 to facilitate clear presentation of the results and consider rationalizing the number of figures, focusing on those presenting novel findings more directly related to the stated objective. The rest would sit nicely in the supplementary information.

 Minor comments:

 Perhaps define ‘ceiling effects’ for the uninformed reader.

 Why were there missing socio-economic data for 29 participants? Were all socio-demographic characteristics missing? Or a proportion? If the latter, how does this group compare with those that completed all of the survey?

 Whilst the authors mention the differences in age and gender between the sample and the general population data, no mention is made regarding education level or marital status. How did the authors determine comparability between the populations?

I confirm that I have read this submission and believe that I have an appropriate level of expertise to confirm that it is of an acceptable scientific standard, however I have significant reservations, as outlined above.

# Peer review: Reviewer response for version 1

**DOI:** 10.21956/hrbopenres.13910.r26392

**Published:** 2018-10-29

**Authors:** Fredrick Dermawan Purba

**Affiliations:** 1 Faculty of Psychology, Universitas Padjadjaran, Bandung, Indonesia

This manuscript provides Ireland population HRQOL norm data measured by the EQ-5D-5L based on a national representative sample. The manuscript is well written, although in my opinion is longer than usual report. Below are some comments to further improve the manuscript:

1. The crude sum score analysis of health states is not the (usually) recommended way to analyze the descriptive part of the EQ-5D-5L. If so, please provide the reference where this is ever done.
2. The index score based on national tariff is common to be reported in a population norm report. The authors should consider doing the same.
3. Paragraph 3 Introduction section: the sentence “…showing broadly consistent pattern…” probably better placed in Discussion section instead of Introduction.
4. Paragraph 2 of Methods-Sample: the sentence “On completion of the random sampling… in the sample” probably better placed in the Discussion section.
5. Paragraph 2 of Methods-Sample: the sentence “A quality control process… of the study” could be skipped since this is more related to the valuation study instead of population norm, since no QC indicator for the descriptive part of the EQ.
6. Some parts of the Methods section: e.g. total sample of 1131, references to Table 1-6 in the text, should be placed in the Results section.
7. Page 5 last paragraph: Please provide details of weighted for age and gender process.
8. Table 1: Formal tests comparing the final sample and general population proportions for each socio-demographic should be conducted.
9. Table 2 and 3: The authors presented the age and gender in Table 3, why bother to do the same with only age in table 2? Or better do it also for gender only then combine the two.

I confirm that I have read this submission and believe that I have an appropriate level of expertise to confirm that it is of an acceptable scientific standard, however I have significant reservations, as outlined above.

# Peer review: Reviewer response for version 1

**DOI:** 10.21956/hrbopenres.13910.r26391

**Published:** 2018-10-17

**Authors:** Pedro Lopes Ferreira

**Affiliations:** 1 Faculty of Economics, Center of Study and Research in Health (CEISUC), University of Coimbra, Coimbra, Portugal

This paper by Hobbins et al addresses the creation of norms regarding the measurement instrument EQ-5D-5L in Ireland. I really enjoyed reading this paper. It is very up-to-date and, to my knowledge, the result of an investigation of excellent quality.

 However, in spite of this quality, I raise some suggestions that I hope will better the paper.

- In the paper, the authors should emphasize that EQ-5D is a generic measure to assess self-perception of health status and not aimed to measure population health. Only in the conclusions the authors refer to self-reported health.
- Looking at EuroQol “official” description of the EQ-5D the word ‘dimension’ is used rather than ‘domain’. I would propose the authors to maintain the word ‘dimension’.
- The misery index (i.e. the sum of the scores obtained by the 5 dimensions) can be seen as a proxy for the severity of the corresponding health status. I am not sure whether this index can be used to describe health status.
- Regarding the sample collection, I did not quite understand why the authors need to recruit an additional set of 102 respondents. An explanation is needed here.
- Figures 1 to 4 show percentages by several sociodemographic variables at each level of each domain. However, three is no mention regarding the statistical significance of the results.

In summary, I reiterate my satisfaction with this article.

I confirm that I have read this submission and believe that I have an appropriate level of expertise to confirm that it is of an acceptable scientific standard.

# Peer review: Reviewer response for version 1

**DOI:** 10.21956/hrbopenres.13910.r26362

**Published:** 2018-10-03

**Authors:** David G.T. Whitehurst, Helen McTaggard-Cowan

**Affiliations:** 1 Faculty of Health Sciences, Simon Fraser University, Burnaby, BC, Canada; 2 Centre for Clinical Epidemiology and Evaluation, Vancouver Coastal Health Research Institute, Vancouver, BC, Canada; 3 BC Cancer Agency, Vancouver, BC, Canada

The paper by Hobbins and colleagues describes population norms for Ireland for the EQ-5D-5L descriptive system. Overall, the study is well written. The analyses and results sections are described in detail (albeit in an atypical order). The study findings are discussed well relative to the current literature. Below are a few suggestive comments for the authors to address to improve the overall quality of the submission.

1. The beginning of the title is a little misleading because the EQ-5D-5L does not measure ‘health’. Consider dropping, “ *The health of the residents of Ireland:*”. The same applies to multiple references in the abstract and introduction (“ *describe the health*”, “ *describes health*”).
2. The paper needs much more justification for the use of the sum score in the analysis. Sum scores (sometimes referred to as a ‘misery index’ or ‘unweighted score’, depending on the calculation method) can be calculated in multiple ways. Is there a sum score that is recommended by the EuroQol Group? If not, what is the justification for the chosen approach and, more importantly, what does analysis of your sum score add to the paper (if it’s a method that is not used very often)?
3. The descriptions of the two different scoring approaches (sum score and preference-weighting) is misleading… they get ‘equal billing’ despite that fact that preference-weighting is more common/relevant.
4. If the sum score remains in the paper, please provide a very explicit statement as to why index scores are not used in this analysis. What is ‘good’ about sum scores in this context? What is ‘bad’ about index scores in this context?
5. Again, if the sum score remains in the paper, a numerical example of the scoring procedure is likely to be helpful for some readers (e.g., explain how health state 13232 = 70).
6. It would be helpful for the readers to fully understand where this current study fits in the larger Irish EQ-5D-5L valuation study. Specifically, when do the respondents complete the tasks for the EQ-5D-5L valuation study and when do the respondents complete the EQ-5D-5L descriptive system? Could the authors briefly discuss the possibility of a framing effect if respondents viewed a series of severe health states prior to self-reporting their own health and how this may affect the population norms?
7. The authors discuss that an additional 102 responses were collected to accommodate the over-representation of study sample. How was this number decided upon? At first glance, n=102 does not appear to be enough respondents to accommodate the over-sampling of females and older people. The provision of test statistics in Table 2 would be helpful to compare the proportion of individuals in the study sample with respect to national data.
8. In the abstract, it is stated that “a usable sample of 1,131 individuals...”. Can you please clarify what “usable” means here? For example, did you exclude respondents if there were missing responses to the EQ-5D-5L? Did you only include respondents if they completed both the EQ-5D-5L and the sociodemographic questions? Any criteria used to determine whether or not an individual was included in the analysis should be described in the Methods section.
9. More information about the weighting process used would be helpful, even if only included as an appendix. Specifically, what source of the population-level data was used in the weighting process?
10. Please change EQ-5D-5L domains to EQ-5D-5L dimensions to reflect the terminology used in the EQ-5D-5L User Manual.
11. Please avoid use of ‘perfect health’ (‘full health’ is more appropriate).
12. Figures would benefit from more informative axes labels and titles.
13. Results are first reported in the Methods section (e.g., the sample size, participant demographics, Tables, etc.). These are not *methods*; please move these details to the Results section.

We confirm that we have read this submission and believe that we have an appropriate level of expertise to confirm that it is of an acceptable scientific standard, however we have significant reservations, as outlined above.
